# Decoupling of Mechanical and Transport Properties in Organogels via Solvent Variation

**DOI:** 10.3390/gels7020061

**Published:** 2021-05-21

**Authors:** Kenneth P. Mineart, Cameron Hong, Lucas A. Rankin

**Affiliations:** Department of Chemical Engineering, Bucknell University, Lewisburg, PA 17837, USA; ch052@bucknell.edu (C.H.); lar026@bucknell.edu (L.A.R.)

**Keywords:** organogel, block copolymer, structure–property relationships, transport, mechanics

## Abstract

Organogels have recently been considered as materials for transdermal drug delivery media, wherein their transport and mechanical properties are among the most important considerations. Transport through organogels has only recently been investigated and findings highlight an inextricable link between gels’ transport and mechanical properties based upon the formulated polymer concentration. Here, organogels composed of styrenic triblock copolymer and different aliphatic mineral oils, each with a unique dynamic viscosity, are characterized in terms of their quasi-static uniaxial mechanical behavior and the internal diffusion of two unique solute penetrants. Mechanical testing results indicate that variation of mineral oil viscosity does not affect gel mechanical behavior. This likely stems from negligible changes in the interactions between mineral oils and the block copolymer, which leads to consistent crosslinked network structure and chain entanglement (at a fixed polymer concentration). Conversely, results from diffusion experiments highlight that two penetrants—oleic acid (OA) and aggregated aerosol-OT (AOT)—diffuse through gels at a rate inversely proportional to mineral oil viscosity. The inverse dependence is theoretically supported by the hydrodynamic model of solute diffusion through gels. Collectively, our results show that organogel solvent variation can be used as a design parameter to tailor solute transport through gels while maintaining fixed mechanical properties.

## 1. Introduction

Traditionally, organogels have been applied in areas wherein their mechanical and chemical properties are beneficial. A few examples include consumer cushioning [1,2,3], model human tissue (for ballistics testing and mock surgery) [4,5], dielectric elastomers [6,7], and hydrophobic filler in underwater cables [8,9]. Recently, aliphatic solvent-based organogels have been considered for use as transdermal drug delivery media due to their relative inertness and inherent adhesiveness [10,11,12,13]. Successful transdermal delivery gels rely upon the controlled release of payload compounds from the gel to the skin, where they can enter the body and perform their designed function. While gel mechanical and chemical properties are still important considerations for transdermal media, understanding the transport of payload compounds through gels is also imperative. 

One specific class of organogels considered for transdermal delivery media are those comprised of styrenic triblock copolymer (e.g., poly[styrene-b-(ethylene-co-butylene)-b-styrene] (SEBS)) and aliphatic gel solvent (selective for the copolymer midblock), which have been previously referred to as thermoplastic elastomer gels [14,15,16]. The styrenic triblock copolymers in these systems self-assemble due to the chemical incompatibility of their S (i.e., polystyrene) endblocks and aliphatic midblock (e.g., poly[ethylene-*co*-butylene] (EB)). The resultant gel structure is a physically-crosslinked network of discrete, glassy S domains (radius ~10 nm) interconnected by rubbery aliphatic midblocks. The copolymer midblocks and midblock-selective gel solvent form a continuous phase surrounding the S endblock crosslinks and macroscopic payload diffusion can occur within this continuous phase. In an earlier study, Ma et al. showed that increasing the amount of styrenic triblock copolymer in gels translates to a decrease in testosterone release rate [10]. They also determined that the aliphatic solvent composition, consisting of aliphatic oil and hydrocarbon tackifying resin, affects the release rate of testosterone. Specifically, increasing the amount of tackifying resin decreased testosterone release rate. The latter result highlights a key advantage of organogels over hydrogels in formulation of transdermal delivery media: the solvent can be tailored to alter gel properties. Our work on SEBS/mineral oil gels agrees with the former result of Ma et al. and further concluded that SEBS concentration simultaneously increases gel stiffness and decreases payload release rate [17].

A more thorough understanding of gel solvent variation is required to take full advantage of solvent variation in the design of organogel delivery media. As a first step, the present study examines the role of solvent viscosity on gel properties. Multiple aliphatic mineral oils with a wide range of dynamic viscosity were incorporated into SEBS-based gels, and the mechanical properties and payload release behavior of these gels was characterized. We hypothesized that alteration of solvent viscosity could be used to decouple gels’ quasi-static mechanical behavior and the release rate of payload. Our results demonstrate that payload release rate, as described by diffusivity of payload through gels, is inversely related to gel solvent viscosity. The inverse relation between payload diffusivity and viscosity is theoretically supported by the hydrodynamic model for solute diffusion through a gel medium. Additionally, collected data confirm our hypothesis: gel viscosity modulation, at a fixed SEBS concentration, changes payload release, but has a negligible effect on gel mechanical behavior.

## 2. Results and Discussion

### 2.1. Solvent Properties

As alluded to above, this study focuses on the effect of varying organogel solvent (i.e., aliphatic mineral oil—MO) on gel properties. It is important to establish the foundational pure solvent properties of each MO prior to discussing gel properties. First, each MO should be midblock selective (i.e., EB selective) such that polystyrene endblocks can aggregate and subsequently vitrify into physical crosslinks. Should the MO have an affinity for polystyrene domains, these domains will be plasticized and gels will not be load-bearing materials. This attribute is typically accomplished through use of aliphatic hydrocarbon solvents. The present MOs, which include squalane (SQUAL), Hydrobrite 200 (HB 200), Hydrobrite 380 (HB 380), Hydrobrite 550 (HB 550), and Hydrobrite 1000 (HB 1000), are all advertised as primarily aliphatic hydrocarbon oils. To confirm this assertion, FTIR spectra were collected for each MO (Figure S1). These spectra all exhibit two weak peaks at ca. 1375 and 1460 cm^−1^ as well as a series of stronger peaks at ca. 2825–3000 cm^−1^. The latter arise from alkane C–H stretching and the former from methyl and methylene C–H bending, respectively. Combined with the absence of peaks that would be expected for other functional groups such as aromatic rings (i.e., 1650–2000 cm^−1^), these results confirm that the MOs used are primarily aliphatic hydrocarbons and should be EB selective.

**Figure 1:**
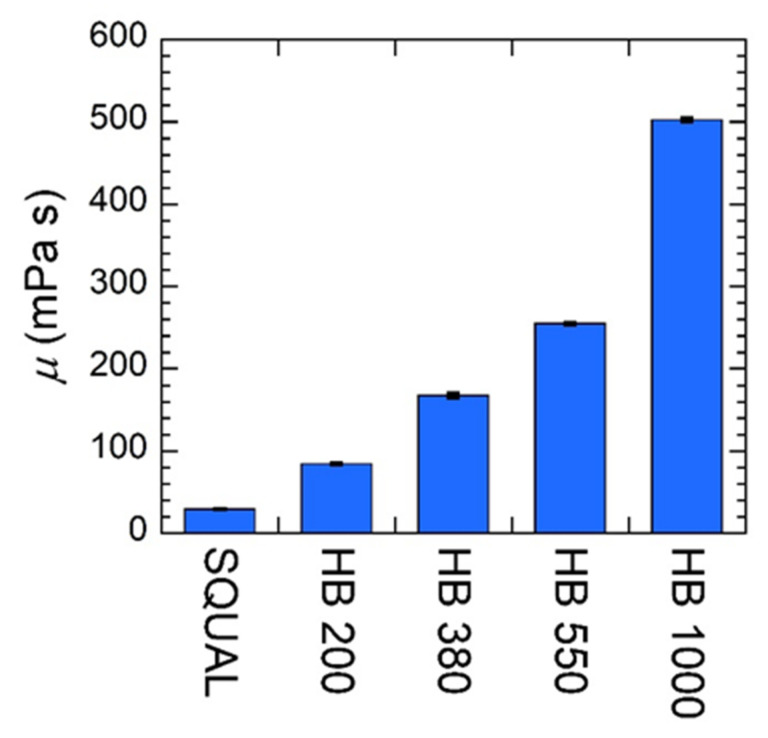
Dynamic viscosity values for various gel solvents used in this study; SQUAL = squalane, HB 200 = Hydrobrite 200, HB 380 = Hydrobrite 380, HB 550 = Hydrobrite 550, and HB 1000 = Hydrobrite 1000.

The second solvent attribute of interest in this study is MO viscosity because it is hypothesized to have an effect on transport within gels. Specifically, the Stokes–Einstein Equation describes the relationship between diffusivity of a penetrant through pure solvent, *D_0_*, and the solvent’s dynamic viscosity, *μ*, as

(1) $$D_{0}=\frac{k_{B}T}{6\pi r_{h}\mu }$$

where *k_B_* is the Boltzmann constant, *T* is temperature, and *r_h_* is the hydrodynamic radius of the penetrant. While the chemical functionality of the MOs is uniform across the grades examined, viscosity is sensitive to the molecular weight and architecture of the compounds that comprise each MO. Thus, the viscosities may vary. Dynamic viscosities measured for each of the five MOs highlight that this factor is prevalent for these MOs (Figure 1). In fact, there is a relatively broad range of dynamic viscosities from SQUAL up to HB 1000, which theoretically translate to a large variation of penetrant diffusion through pure solvent: *D_0,SQUAL_*/*D_0,HB 1000_* ≈ 20.

### 2.2. Gel Preparation

In order to understand the effect of gel solvent viscosity on gel properties, organogels were fabricated using each of the MOs along with SEBS copolymer (number-average molecular weight, *M_n_* = 125 kDa, polystyrene fraction, *f_S_* = 0.33 g polystyrene/g, and polydispersity *Ð* = 1.01) as the gelator and either oleic acid (OA) or aerosol-OT (AOT) as the penetrant compound. Oleic acid resides in SEBS/MO gels as individual molecules (i.e., unimers), whereas aerosol-OT aggregates into reverse micelles with a radii of ca. 1.7 nm. Gels were initially formulated with 10 wt%, 20 wt%, or 30 wt% copolymer and 0.5 wt% OA or 1.0 wt% AOT (values based on previous work [17,18]), resulting in six series (in each series, the MO identity was varied). Note, 1.0 wt% AOT loading resulted in cloudy gels for HB 550 and HB 1000 MOs, suggesting that precipitation occurred. As a result, only SQUAL, HB 200, and HB 380 were considered when AOT was present. All gels were preswollen prior to any experimentation in a solution-matched liquid (e.g., a 10 wt% SEBS/0.5 wt% OA/89.5 wt% HB 380 gel was preswollen in a 0.5 wt% OA/99.5 wt% HB 380 solution) until equilibrium swollen mass was achieved. This process causes the concentration of SEBS to decrease due to an increase in MO, but the penetrant concentration remains fixed. Table 1 provides a summary of the final gel formulations. In the remainder of our discussion, we refer to each of the copolymer concentration sets by their average concentrations: 6.5 wt%, 11.2 wt%, and 15.7 wt%.

### 2.3. Mechanical Behavior

**Figure 2:**
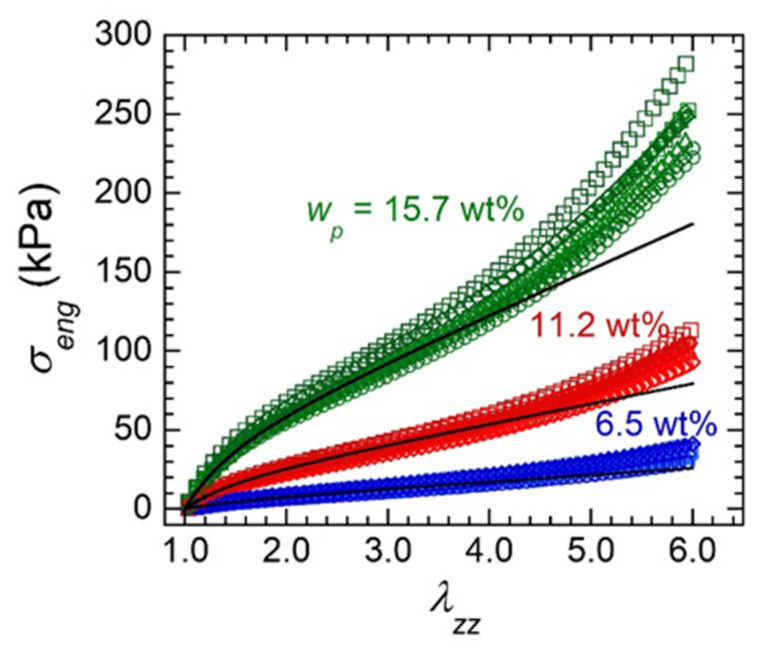
Representative stress–stretch profiles for gels containing various oils (circles = squalane, squares = HB 200, diamonds = HB 380, triangles = HB 550, crosses = HB 1000) at different SEBS concentrations (labeled) with either 0.5 wt% OA (light symbols) or 1.0 wt% AOT (dark symbols). Lines indicate a representative STN model fit for each group of fixed SEBS concentration gels.

Quasi-static, uniaxial tensile experiments were conducted for each gel batch shown in Table 1 from *λ_zz_* = 1 to *λ_zz_* = 6, where *λ_zz_* is the stretch ratio defined by the length at a given time, *L*, to that at the start of the experiment, *L_0_* (i.e., *L*/*L_0_*). Two qualitative observations are apparent from engineering stress, *σ_eng_*, vs. stretch data across all 24 gel batches (Figure 2). First, full stress–stretch profiles are closely bunched together for a fixed SEBS concentration across all MO and penetrant identities (as indicated by similar colors in Figure 2). Second, the stress at any given stretch ratio increases with SEBS concentration implying gels become stiffer as a consequence of increasing polymer concentration.

**Figure 3:**
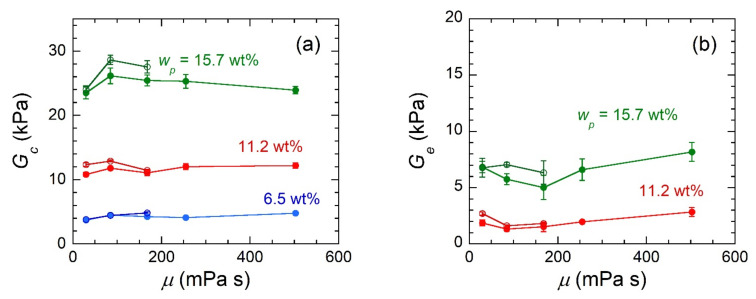
(**a**) Crosslinked network modulus contribution for gels composed of 0.5 wt% OA (filled symbols) or 1.0 wt% AOT (open symbols) and varying SEBS concentration (labeled) and oil viscosity. (**b**) Chain entanglement modulus contribution for gels composed of 0.5 wt% OA (filled symbols) or 1.0 wt% AOT (open symbols) and varying SEBS concentration (labeled) and oil viscosity. Lines serve as guides to the eye.

Quantitative mechanical characteristics can be acquired by fitting stress–stretch profiles with an applicable model. The physically-crosslinked network structure of SEBS gels is amenable to fitting with a revised form of the slip-tube network (STN) model [19,20], which includes a correction for the filler effect imparted on gels’ mechanical properties by the glassy polystyrene crosslink domains

(2) $$\sigma_{eng}=\left(G_{c}+\frac{G_{e}}{0.74\lambda_{zz}+0.61\lambda_{zz}^{-0.5}-0.35}\right)\left(\lambda_{zz}-\lambda_{zz}^{-2}\right)\left(1+2.5\phi_{PS}+14.1\phi_{PS}{}^{2}\right)$$

where *G_c_* and *G_e_* are the modulus contributions from the crosslinked network and the entangled EB midblocks, respectively, and *ϕ_PS_* is the volume fraction of polystyrene in the gel. The values of *ϕ_PS_* for each gel batch are calculated from known information, namely *ϕ_PS_* = *f_S_* × *w_SEBS_*(*ρ_S_*/*ρ_g_*) where *f_S_* is the fraction of polystyrene in SEBS, *w_SEBS_* is the SEBS concentration in the gel, and *ρ_S_* and *ρ_g_* are the densities of polystyrene and the gel, respectively. As a result, *G_c_* and *G_e_* are the only fitting parameters used to describe stress–stretch data with the STN model and the fitted model represents data well up to *λ_zz_* ≈ 4 as has been noted in previous studies [17,19,21]. Furthermore, the values of *G_c_* and *G_e_* determined through fitting match the observations discussed above. Penetrant and MO identity have minimal effect on *G_c_* and *G_e_*, whereas both modulus contributions increase dramatically with increasing SEBS concentration (Figure 3). The minor fluctuations observed for *G_c_* and *G_e_* when penetrant and MO identity are varied likely arise as a result of small differences in *w_SEBS_* across these gels. These results highlight that the properties of the solvent alone do not affect gels’ quasi-static mechanical behavior so long as the solvent-polymer interactions remain approximately the same. It also shows that the presence of penetrants in either form does not impact quasi-static mechanical response.

Deeper exploration of the modulus contribution parameters allows these results to be further understood. The affine network definition of *G_c_* is

(3) $$G_{c}=\nu k_{B}T$$

where *ν* is the number density of networked EB chains within gels, and is the only parameter in this expression that varies across gel systems, here. It is expected that *ν* is only a function of *w_SEBS_* because the amount of copolymer in gels directly affects the number density of EB blocks, but solvents with similar functionality should not affect this parameter. This interpretation agrees with the trends seen in Figure 3a. Alternatively, *G_e_* is typically described as a power-law proportional to *w_SEBS_*, wherein the exponent depends on the quality of the solvent for the polymer (i.e., *G_e_* ∝ *w_SEBS_*^2.25^ for a good solvent or *G_e_* ∝ *w_SEBS_*^2.33^ for a theta solvent) [20,22,23]. We expect that *G_e_*, like *G_c_*, is only dependent on *w_SEBS_* and not *μ* since the chemical functionality of the MOs is relatively unchanged. This interpretation agrees with the trends seen in Figure 3b. There is clear experimental and theoretical dependence of *G_c_* and *G_e_* on *w_SEBS_*; however, these relationships are not explored in detail at present because the focus of this study is on the effect of oil viscosity on gel properties and because of the considerable complexity of polymer chain conformation that exists in gels formulated at different *w_SEBS_* followed by post-anneal swelling.

### 2.4. Transport Properties

In contrast to gel mechanical behavior, we hypothesize that the transport of penetrants through gels should be affected by MO viscosity. Penetrant (i.e., OA or AOT) release experiments were conducted by submersing gels into pure MO (the identity of the oil matched that of the gel under observation) and monitoring the relative amount of OA or AOT retained within each gel over time. OA and AOT concentrations were measured using a previously described FTIR-based method that takes advantage of the isolated peak position of each penetrant compound’s ester/acid carbonyl group(s) (OA = 1712 cm^−1^, AOT = 1739 cm^−1^) [18]. Examples of the time evolution of FTIR spectra during release experiments are shown in Figure S2 (OA) and Figure S3 (AOT) and relative retained mass (i.e., *m*/*m_0_* where *m* and *m_0_* are penetrant mass in a gel at time *t* and time *0*, respectively) corresponds directly to relative peak absorbance (*A*/*A_0_* where *A* and *A_0_* are carbonyl peak absorbance at time *t* and time *0*, respectively).

**Figure 4:**
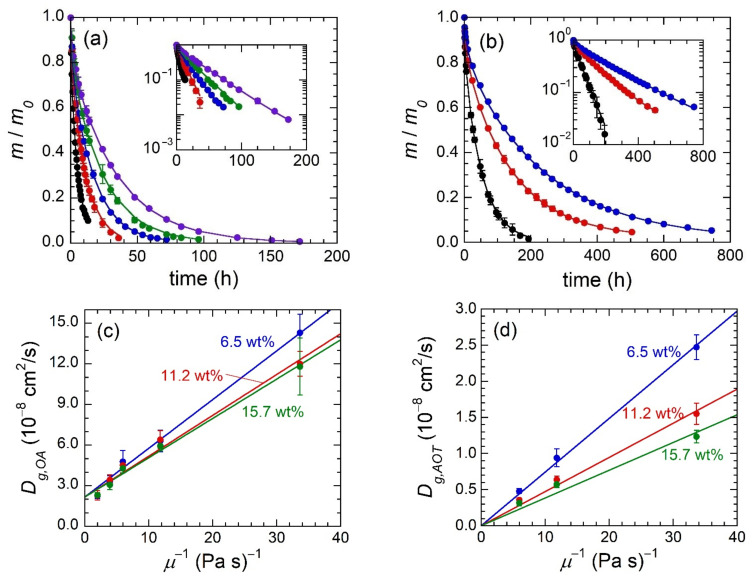
Time-resolved retained mass of OA (**a**) and AOT (**b**) for gels comprised of various MOs (black = squalane, red = HB 200, blue = HB 380, green = HB 550, purple = HB 1000) and 11.2 wt% SEBS. Insets show the same data plotted on a semi-log scale. Diffusivity values for OA (**c**) and AOT (**d**) through gels composed of varying SEBS concentration (labeled) and MO identity. Lines in (**a**) and (**b**) are fits using Equation (5), whereas lines in (**c**) and (**d**) are linear fits to the data including a fixed y-intercept value of 0 cm^2^/s in (**d**).

Examination of OA and AOT retained mass profiles (Figure 4a,b) clearly shows that increasing MO viscosity leads to slower release of the penetrant compound. In gels containing OA, the time required for 90% penetrant release ranges from ≈10 h for the lowest viscosity MO (SQUAL) to ≈72 h for the highest viscosity MO (HB 1000). Similarly, gels that contain AOT range in the time required for 90% penetrant release from ≈120 h (SQUAL) to ≈600 h (HB 380). Each OA, or AOT, retained mass profile can be modeled to quantify penetrant diffusivity through gels—*D_g,i_*, where *i* is either OA or AOT. Fick’s Second Law of Diffusion for a disk is

(4) $$\frac{\partial C_{i}}{\partial t}=D_{g,i}\left[\frac{1}{r}\frac{\partial }{\partial r}\left(r\frac{\partial C_{i}}{\partial r}\right)+\frac{1}{r^{2}}\left(\frac{\partial^{2}C_{i}}{\partial \theta^{2}}\right)+\frac{\partial^{2}C_{i}}{\partial z^{2}}\right]$$

where *C_i_* is the concentration of penetrant in the gel, *r*, *θ*, and *z* are radial, angular, and axial coordinates, and *t* is time. This expression can be simplified by assuming that diffusion occurs predominantly in the axial direction of gels due the absence of a concentration gradient in the *θ* direction and their geometry (radius ≈ 10 × thickness), which eliminates the first and second terms in the square brackets. The simplified form of Equation (4) can be solved by applying one initial condition (the penetrant concentration is uniform in gels at *t* = 0) and two boundary conditions (the penetrant concentration gradient at the center of gels is zero and the liquid oil bath provides an effective concentration sink). Subsequent integration of this solution over the full gel thickness yields

(5) $$\frac{m}{m_{0}}=\frac{8}{\pi^{2}}\exp\left(-\frac{\pi^{2}D_{g,i}}{4L^{2}}t\right)$$

where *L* is half the gel thickness. Modeling of the retained mass profiles in Figure 4a,b using Equation (5) only requires fitting of *D_g,i_* since gel thickness is known. The resultant model fits represent retained mass profiles very well as seen most clearly by retained mass versus time semi-log plots (Figure 4a,b insets).

Diffusivity values extracted through fitting of the data in Figure 4a,b can now be used to directly compare the rate of penetrant transport between gels comprised of different MOs and SEBS concentrations (Figure 4c,d). First, it can be seen that MO viscosity has a strong influence on the diffusivity of both penetrants and their trend qualitatively matches the Stokes–Einstein Equation, which states that *D_g,i_* ∝ *μ*^−1^. Second, *w_SEBS_* has a small effect on OA diffusivity and a moderate effect on AOT diffusivity, but in both cases the impact of *w_SEBS_* is considerably smaller than *μ*.

We set out to further understand the relationship between diffusivity and formulation parameters by theoretically describing our data with an established model for solute diffusion through a polymeric gel medium. Specifically, we elect to use the hydrodynamic description of solute diffusion as it aligns most directly with the Stokes–Einstein Equation. The hydrodynamic description of solute diffusion treats the penetrant as a hard sphere moving at a constant velocity through the gel medium. The hard spheres’ motion is hindered by frictional drag within the gel stemming from the properties of the solvent and the concentration and properties of the polymer chains. The hydrodynamic model derived for penetrant diffusion through homogenous gels [24,25] takes the form

(6) $$D_{g,i}=\left[\frac{k_{B}T}{6\pi r_{h,i}}\left(\frac{1}{\mu }\right)\right]\exp\left(-r_{h,i}k_{c}\phi_{p}^{3/4}\right)$$

where *k_c_* is a polymer/solvent-specific constant, *ϕ_p_* is the polymer volume fraction, and the penetrant hydrodynamic radii are *r_h,OA_* ≈ 5 Å (unimers) and *r_h,AOT_* ≈ 17 Å (reverse micelles). The term in square brackets of Equation (6) reflects frictional drag at infinite polymer dilution (i.e., *ϕ_p_* = 0) and can, therefore, be thought of as the solvent contribution to diffusivity. Alternatively, the exponential term of Equation (6) arises from the frictional drag associated with polymer chains within gels.

It is important to reframe the definition of *ϕ_p_* in Equation (6) in the context of the current systems before proceeding. Because penetrant molecules are excluded from the crosslink domains of the present organogels, their diffusion only occurs in the MO-rich continuous phase. Therefore, *ϕ_p_* is more specifically defined as the volume fraction of EB midblocks in the EB/MO continuous phase. This new definition yields

(7) $$\phi_{p}=\frac{V_{EB}}{V_{EB/MO}}=\frac{\left\{\left(1-f_{S}\right)\;w_{SEBS}\right\}/\rho_{EB}}{\left\{1-\left(f_{S}\;w_{SEBS}\right)\right\}/\rho_{EB/MO}}$$

where *V_EB_* and *V_EB/MO_* are the volumes of EB midblocks and of the EB/MO continuous phase in gels, respectively, and *ρ_EB_* and *ρ_EB/MO_* are the densities of the EB midblocks (0.878 g/cm^3^) and of the EB/MO continuous phase (≈0.861 g/cm^3^), respectively. For reference, approximate values of *ϕ_p_* for 6.5 wt% SEBS, 11.2 wt% SEBS, and 15.7 wt% SEBS gels are 4.4 vol%, 7.6 vol%, and 10.9 vol%, respectively.

With Equation (6) and corresponding considerations in place, we now revisit the experimental data in Figure 4c,d. If the relationship between *D_g,i_* and *μ* is accurately described by Equation (6), the trends of *D_g,i_* versus *μ*^−1^ should be linear with a *y*-intercept of zero. In all six cases (three SEBS concentrations and two penetrant identities), linear fits describe data well and in the case of AOT diffusion the fit can be forced through a *y*-intercept of 0 cm^2^/s while maintaining a good fit to the data (Figure 4d). OA diffusion, on the other hand, is clearly not amenable to fitting with a *y*-intercept of zero. However, all three series roughly converge to a common *y*-intercept of ca. 2.2 × 10^−^^8^ cm^2^/s. (This point will be addressed further below). The slope of each individual linear fit, which has a fixed *r_h,i_* and *ϕ_p_*, should correspond to [*k_B_T*/(6π *r_h,i_*)]exp(−*r_h,i_ k_c_ ϕ_p_*^3/4^), wherein only *k_c_* is unknown, but should remain constant across all data series. Minimizing the squared difference between experimental and theoretical slopes (Table 2) results in *k_c_* = 0.39 Å^−1^, which is of appropriate magnitude based on previous studies [25].

The collection of evidence in the preceding paragraph mostly describes the experimental penetrant transport findings. The only point that does not agree with the hydrodynamic theory is the non-zero *y*-intercept of the three gel series that contain OA as the penetrant. The non-zero *y*-intercept suggests that diffusion of OA molecules will occur in gels comprised of MO with infinite viscosity. In contrast, the AOT diffusivity values match expectation; the diffusivity approaches zero as viscosity approaches infinity. One possible explanation is that a fraction of OA diffusion occurs through hopping of OA molecules amongst the free volume voids between MO and EB molecules (referred to as free volume theory) [25,26]. Even when the solvent viscosity approaches infinity and the fraction of OA diffusion occurring based on the hydrodynamic description becomes negligible, the OA molecules diffuse via the free volume sites. On the other hand, the AOT reverse micelles are too large to hop among free volume voids and are fully described by the hydrodynamic diffusion model.

## 3. Conclusions

Herein, we have presented our experimental results from block copolymer organogels formulated with various MOs. These MOs have similar chemical functionality, but different dynamic viscosities. Our data show that MO viscosity has a negligible role in gels’ quasi-static mechanical behavior owing to the fact that they do not contribute to, or have an effect on, gels’ physically-crosslinked network or polymer chain entanglement. Alternatively, MO viscosity strongly affects the rate of penetrant diffusion through gels. These findings highlight one major benefit of organogels over hydrogels in transport applications: gel mechanical and transport properties can be independently tuned through judicious gel solvent variation.

## 4. Materials and Methods

### 4.1. Materials 

Block copolymer organogels were all fabricated using poly[styrene-*b*-(ethylene-*co*-butylene)-*b*-styrene] (SEBS) produced by Kraton Polymers LLC, Houston, TX, USA (G1654 grade, *M_n_* = 125 kDa, *f_S_* = 0.33 g S/g, *Ð* = 1.01). Aliphatic oils used as the gel solvent included squalane (98% pure, Alfa Aesar, Ward Hill, MA, USA) and various grades of Hydrobrite^®^ from Sonneborn LLC, Petrolia, PA, USA (HB 200, HB 380, HB 550, and HB 1000). Additionally, oleic acid (OA, >99% pure, TCI America, Portland, OR, USA) or aerosol-OT (AOT, >97% pure, Sigma Aldrich, St. Louis, MO, USA) was incorporated into gels for diffusion measurements. Toluene (>99.5% pure, VWR, Radnor, PA, USA) was used as the common solvent in gel preparation.

Organogels were prepared by dissolving the desired quantities of SEBS, oil, and AOT (or OA) in toluene at a 1:20 mass:volume ratio. For example, 2.0 g SEBS, 7.9 g squalane, and 0.1 g AOT were dissolved in 200 mL of toluene. Upon complete mixing as ascertained from a clear homogeneous solution, mixtures were rotary evaporated to remove toluene. Resultant gel products were annealed in a vacuum oven (120 °C, 0.95 atm) for >18 h and then melt-pressed (120–160 °C, minimal applied pressure) into the desired geometry. Finally, gels were preswollen to their equilibrium mass in a solution matching their gel solvent composition (e.g., for a gel composed of SEBS, squalane, and 1 wt% AOT, the preswelling solution was squalane with 1 wt% AOT). Gels that were initially formulated with 10 wt%, 20 wt%, and 30 wt% SEBS had final average SEBS concentrations of ca. 6.5 wt%, 11.2 wt%, and 15.7 wt%, respectively.

### 4.2. Viscosity Measurements

The viscosity of the various gel solvents was measured on a Brookfield (Middleboro, MA, USA) DVE viscometer with small sample adapter (6.7 mL). The spindle speed and equilibration time for each measurement varied based on the solvent under analysis: 100 rpm/1 min (squalane), 30 rpm/2 min (HB 200), 12 rpm/3 min (HB 380), 10 rpm/4 min (HB 550), and 5 rpm/5 min (HB 1000). Measurements were repeated in triplicate to ensure reproducibility.

### 4.3. FTIR Measurements

Fourier-transform infrared spectroscopy (FTIR) was performed on a Thermo Scientific (Waltham, MA, USA) Nicolet iS10 spectrometer at ambient temperature and under N_2_ purge. All final spectra were collected at a resolution of 0.5 cm^−1^ and were averaged based on 32 raw spectra runs. Transmission measurements were collected for solid films using a solid sample holder without any windows. Attenuated total reflectance (ATR) measurements were collected for solutions using a diamond ATR crystal.

### 4.4. Tensile Experiments

Quasi-static uniaxial tensile tests were performed using a single axis on an ADMET (Norwood, MA, USA) eXpert 8000 planar biaxial tester equipped with a 5 lb load cell. All samples were subjected to a constant strain rate of 0.2 mm/s (equivalent of 0.01 s^−1^) from their initial length to 500% strain (*λ_zz_* = 6). Load data were converted to stress by accounting for gel strips’ width and thickness (i.e., cross-sectional area), which ranged from 8.6 to 9.7 mm and 1.6 to 1.9 mm, respectively, depending on copolymer concentration. (Copolymer concentration effects extent of equilibrium preswelling).

### 4.5. Diffusion Experiments

Diffusion experiments were conducted by submersing preswollen gel disks (thickness = 1.6–1.9 mm, diameter = 28.5–33.9 mm, depending on extent of preswelling) containing either 0.5 wt% OA or 1.0 wt% AOT into the corresponding gel solvent in the absence of OA or AOT (e.g., pure squalane). The solvent quantity was held at a fixed ratio of 100 mL per 3 g of gel and all samples (i.e., gel disks submersed in oil) were agitated on a shaker table operated at 200 rpm over the duration of diffusion experiments. Gels were regularly extracted from these solutions for transmission FTIR and gravimetric analysis. The latter validated that no significant swelling of gels occurred during diffusion experiments, whereas the former enabled retained mass profiles to be determined based upon peak absorbance values [18].

## Figures and Tables

**Table 1:** Final SEBS concentrations for each formulated and subsequently preswollen gel batch. Within each box, gels with 0.5 wt% OA and 1.0 wt% AOT appear on the left and right, respectively.

	Formulated SEBS Concentration		
MO Identity	10 wt%	20 wt%	30 wt%
SQUAL	6.8 wt%/7.1 wt%	11.5 wt%/12.0 wt%	16.4 wt%/16.3 wt%
HB 200	5.9 wt%/5.5 wt%	10.2 wt%/10.5 wt%	14.4 wt%/14.9 wt%
HB 380	6.0 wt%/6.6 wt%	10.1 wt%/11.5 wt%	14.4 wt%/15.8 wt%
HB 550	6.5 wt%/–^A^	11.1 wt%/–^A^	15.7 wt%/–^A^
HB 1000	7.1 wt%/–^A^	11.8 wt%/–^A^	16.5 wt%/–^A^
average	6.5 wt%	11.2 wt%	15.7 wt%

^A^ AOT was observed to be insoluble in HB 550 and HB 1000 so these gels were not studied further.

**Table 2:** Comparison between experimentally acquired slopes (see Figure 4c,d) and theoretically calculated values using *k_c_* = 0.39 Å^−1^.

	OA (10^−14^ J/m)		AOT (10^−14^ J/m)	
*w_SEBS_*	Exp.	Theor.	Exp.	Theor.
0.065	36.0	35.5	7.4	6.7
0.115	30.2	32.2	4.7	4.8
0.157	29.1	29.5	3.8	3.5

## Data Availability

Data is contained within the article and Supplementary Materials. The data will be gladly shared in Excel format on request.

## Supplementary Materials

The following are available online at https://www.mdpi.com/article/10.3390/gels7020061/s1, Figure S1: FTIR spectra collected in the ATR mode for various oils used in this study: SQUAL = squalane, HB 200 = Hydrobrite 200, HB 380 = Hydrobrite 380, HB 550 = Hydrobrite 550, and HB 1000 = Hydrobrite 1000. Figure S2: FTIR spectra collected over the duration of release experiments for gels comprised of 11.2 wt% SEBS, HB 380 MO, and OA. Figure S3: FTIR spectra collected over the duration of release experiments for gels comprised of 11.2 wt% SEBS, HB 380 MO, and AOT.
