# Supplementary material for: Decoupling of Mechanical and Transport Properties in Organogels via Solvent Variation
Source: Gels. 2021 May 21;7(2):61. doi: 10.3390/gels7020061 (PMC8162343; doi:10.3390/gels7020061)
Supplement: Supplementary file 1 [file gels-07-00061-s001.zip › gels-1222142-supplementary.pdf]

Supplementary Materials

Decoupling of Mechanical and Transport Properties in Organogels via Solvent Variation

Kenneth P. Mineart *, Cameron Hong and Lucas A. Rankin

Department of Chemical Engineering, Bucknell University, Lewisburg, PA 17837, USA; ch052@bucknell.edu (C.H.); lar026@bucknell.edu (L.A.R.)

* Correspondence: kpm007@bucknell.edu

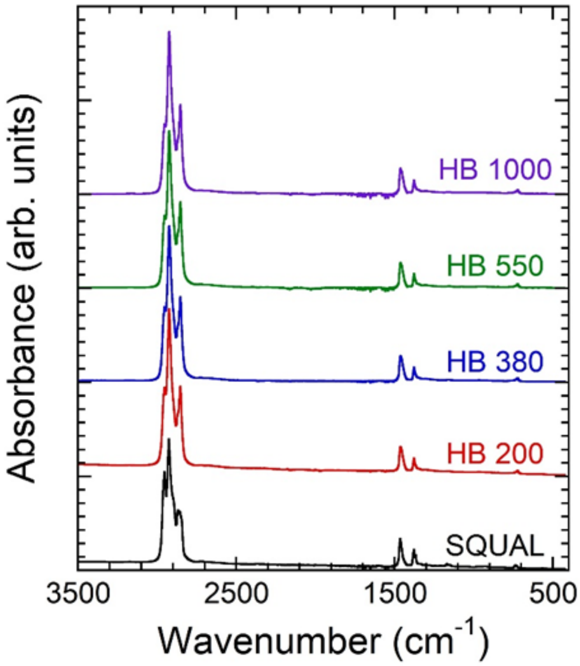

Figure S1. FTIR spectra collected in ATR mode for various oils used in this study: SQUAL = squalane, HB 200 = Hydrobrite 200, HB 380 = Hydrobrite 380, HB 550 = Hydrobrite 550, and HB 1000 = Hydrobrite 1000.

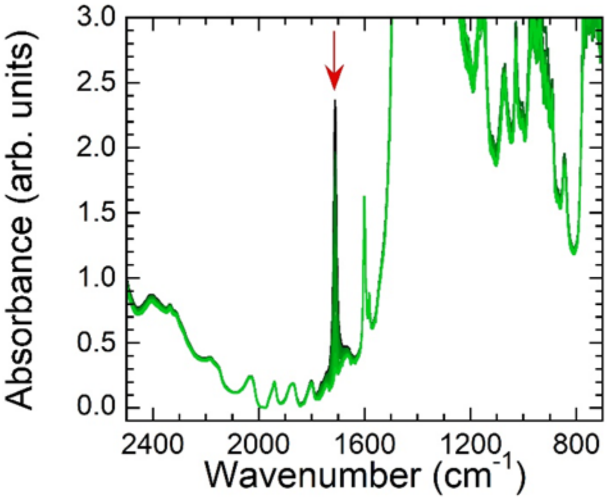

Figure S2. FTIR spectra collected over the duration of release experiments for gels comprised of 11.2 wt% SEBS, HB 380 MO, and OA. The progression from black to light green shows time evolution from 0 h to 72 h and the red arrow indicates the 1712 cm^{-1} peak position of the OA molecule's carbonyl group.

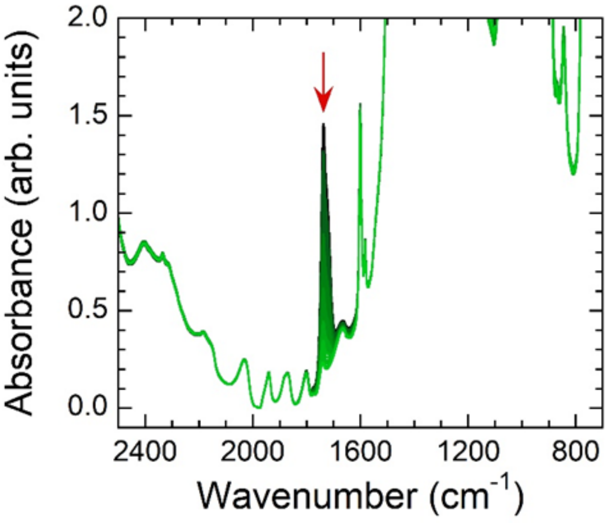

Figure S3. FTIR spectra collected over the duration of release experiments for gels comprised of 11.2 wt% SEBS, HB 380 MO, and AOT. The progression from black to light green shows time evolution from 0 h to 744 h and the red arrow indicates the 1739 cm^{-1} peak position of the AOT molecule's carbonyl groups.
